# Evaluation of Error Production in Animal Fluency and Its Relationship to Frontal Tracts in Normal Aging and Mild Alzheimer’s Disease: A Combined LDA and Time-Course Analysis Investigation

**DOI:** 10.3389/fnagi.2021.710938

**Published:** 2022-01-12

**Authors:** Yoshihiro Itaguchi, Susana A. Castro-Chavira, Knut Waterloo, Stein Harald Johnsen, Claudia Rodríguez-Aranda

**Affiliations:** ^1^Department of Psychology, Keio University, Tokyo, Japan; ^2^Department of Psychology, UiT The Artic University of Norway, Tromsø, Norway; ^3^Department of Neurology, University Hospital North Norway, Tromsø, Norway; ^4^Brain and Circulation Research Group, Department of Clinical Medicine, UiT The Artic University of Norway, Tromsø, Norway

**Keywords:** semantic verbal fluency, perseverations, intrusions, time-course analysis, LDA, frontal tracts, executive dysfunction, mild Alzheimer’s disease

## Abstract

Semantic verbal fluency (VF), assessed by animal category, is a task widely used for early detection of dementia. A feature not regularly assessed is the occurrence of errors such as perseverations and intrusions. So far, no investigation has analyzed the how and when of error occurrence during semantic VF in aging populations, together with their possible neural correlates. The present study aims to address the issue using a combined methodology based on latent Dirichlet allocation (LDA) analysis for word classification together with a time-course analysis identifying exact time of errors’ occurrence. LDA is a modeling technique that discloses hidden semantic structures based on a given corpus of documents. We evaluated a sample of 66 participants divided into a healthy young group (*n* = 24), healthy older adult group (*n* = 23), and group of patients with mild Alzheimer’s disease (AD) (*n* = 19). We performed DTI analyses to evaluate the white matter integrity of three frontal tracts purportedly underlying error commission: anterior thalamic radiation, frontal aslant tract, and uncinate fasciculus. Contrasts of DTI metrics were performed on the older groups who were further classified into high-error rate and low-error rate subgroups. Results demonstrated a unique deployment of error commission in the patient group characterized by high incidence of intrusions in the first 15 s and higher rate of perseverations toward the end of the trial. Healthy groups predominantly showed very low incidence of perseverations. The DTI analyses revealed that the patients with AD committing high-error rate presented significantly more degenerated frontal tracts in the left hemisphere. Thus, our findings demonstrated that the appearance of intrusions, together with left hemisphere degeneration of frontal tracts, is a pathognomic trait of mild AD. Furthermore, our data suggest that the error commission of patients with AD arises from executive and working memory impairments related partly to deteriorated left frontal tracts.

## Introduction

Besides memory complaints, one of the most characteristic dysfunctions in Alzheimer’s disease (AD) is verbal deterioration. Verbal impairments are observed in the quantity, quality, and meaningfulness of verbal response as well as in the ability of verbal comprehension (Lezak, 1995). In particular, AD impairs verbal fluency (VF), which is the ability to generate words as fast as possible according to either a letter of the alphabet or a semantic category within a time limit, usually one minute. VF abilities are evaluated with tasks, such as the Controlled Oral Word Association Test (COWAT, Benton, 1967) for the assessment of phonemic fluency, or by categories, such as animals or supermarket items (see e.g., the Dementia Rating Scale, Mattis, 1976), for the assessment of semantic fluency. The semantic variant of VF has been largely used in the detection of dementia, as the disease causes degradation of the semantic store (Henry et al., 2004). Moreover, it has been reported that AD impairs particularly semantic fluency as compared to phonemic fluency (Laws et al., 2010). Impairments in semantic fluency comprise reduction in the number of correct responses and increment in errors, which are prominent in AD (Gomez and White, 2006) even in the early stages (Fagundo et al., 2008).

From a clinical point of view, identification of errors is of special importance. The reason is that reduced number of generated words occurs in parallel to the occurrence of errors, especially in neuropsychiatric disorders (Suhr and Jones, 1998; Neill et al., 2014). Most common error forms in VF comprise perseverations (i.e., repeated words) and intrusions (i.e., words not pertaining to a semantic category). Perseverations are defined as the continuation or repetition of an action, which has no relevance for the task at hand and becomes unsuitable (Sandson and Albert, 1984). According to an earlier study (Albert, 1989), perseverations are strongly related to neural alterations, and different types of perseverations have been proposed (Sandson and Albert, 1984). Although other forms of errors have been reported in the literature, such as unintelligible or wrong language errors (Roberts and Le Dorze, 1997), for the present investigation, we will only refer to the most common error forms produced in semantic VF, i.e., perseverations and intrusions.

Regarding intrusions, these can be defined as the unintentional recall of an incorrect verbal material for the task demanded (Fuld et al., 1982). Intrusions arise because of deficient lexicon and troubles in retrieval of information from semantic memory (Carlesimo and Oscarberman, 1992). Even if intrusions occur in normal aging (McDowd et al., 2011), a higher propensity of intrusions exist in AD (Hart et al., 1986; McDowd et al., 2011). A classification of intrusions has also been proposed depending on the type of stimuli (Loewenstein et al., 1991).

Perseverations and intrusions in semantic VF are regularly accounted for in the standard scoring of VF by simply reporting the total number of error occurrences (Strauss et al., 2006). Often, the total number of errors (i.e., perseverations + intrusions) are used to calculate a single error score, which is frequently excluded from data analysis (e.g., Rinehardt et al., 2014). However, some investigations report rate of occurrence for perseverations and intrusions separately (e.g., Raboutet et al., 2010). Although error propensity is a low base rate variable (Woods et al., 2004), quantification of total number of perseverations and/or intrusions committed in a minute allows for identification of pathological conditions such as AD (e.g., Ober et al., 1986; Pekkala et al., 2008). This practice has a clinical value. For instance, perseverations in semantic VF have been analyzed in more detail in aging and dementia studies. Pekkala et al. (2008) analyzed a type of perseverations in different VF categories in normal aging, and mild and moderate AD. These authors showed that recurrent and continuous perseverations increase during the course of AD. More recently, Pakhomov et al. (2018) applied longitudinally a computerized solution to assess recurrent perseverations in the “animal” category, which predicted cognitive impairment in a time of 5.5 years. In this line of investigation, Auriacombe et al. (2006) conducted a longitudinal study on a sample of patients with incident AD and age-matched controls. These authors accounted for both perseverations and intrusions to evaluate whether error production in semantic VF characterized different phases of the disease. Indeed, the findings showed that perseverations were a marker of AD at diagnosis time. The abovementioned studies bring out the importance of analyzing errors in semantic VF. However, the purpose and methods employed had clinical interests, and they were not designed to disclose the mechanisms behind error occurrence. In this regard, a search in the literature revealed one investigation by Miozzo et al. (2013) looking at the mechanisms behind perseverations in semantic VF in AD. This study applied a time-analysis approach, which revealed that perseverative answers took place after long lags from their first occurrence. Conclusions were that perseverations emerged because of executive dysfunction and memory deficits.

Although that evaluation of errors (mainly of perseverations) has shown diagnostic utility and promising findings, there is still a need to unveil the significance of both intrusions and perseverations in semantic VF errors in aging and AD. To the best of our knowledge, there are no previous investigations that have attempted to disentangle the underlying mechanisms of different types of errors in the mentioned populations. In fact, the majority of studies in the field uses error data only to obtain an accuracy score, i.e., correct number of generated words, which is the main outcome of semantic VF. Correct answers have been analyzed with different quantitative and qualitative methods. One of the first qualitative approaches was proposed by Troyer et al. (1997) who calculated clustering and switching scores based on correct answers produced. Subsequent studies have introduced time-course analyses (e.g., Demetriou and Holtzer, 2017), and computational (e.g., latent semantic analysis; e.g., Ledoux et al., 2014) and statistical techniques (e.g., multidimensional scaling; e.g., Weakley and Scmitter-Edgecombe, 2014) aiming to ameliorate the qualitative scrutiny of semantic VF performance. These methodologies have proven successful as they allow us to appraise qualitative differences of semantic word generation. Nonetheless, the application of these methods has not been implemented to analyze the errors in semantic VF.

Because the simple report of total number of errors cannot reveal the exact nature of cognitive deteriorations taking place, the present investigation will carry out a refined evaluation of intrusions and perseverations during semantic VF in normal aging and mild AD. The primary purpose is to better understand the underlying cognitive mechanisms of errors. To achieve this objective, the application of different approaches and techniques is deemed necessary. As a first step, a behavioral analysis of the pattern of error generation seems appropriate. With this analysis, we wish to determine how and when semantic VF errors occur. To answer these questions, we need to establish the context in which errors are committed and code the exact time of error occurrence. Defining the context in which errors happen can help answer the “how,” and this can be achieved by identification of *clusters* or bundles of semantically related words (Troyer et al., 1997). This strategy provides a structure of word generation (Zemla et al., 2020). Previous data have demonstrated that clusters in semantic VF significantly affect perseveration rate (Azuma, 2004). Therefore, we wish to evaluate whether the context (i.e., presence of clusters) plays a role in error production of both perseverations and intrusions. Regarding the second question of “when,” we apply time-course analysis techniques to address the issue. In our previous study, we have demonstrated that calculation of in-between word intervals during VF is a relevant way to assess the information processing speed of word generation during VF in aging populations (Rodríguez-Aranda et al., 2016). In the present investigation, we use the same approach to register when errors take place. In addition, we go one step further by not only analyzing perseverations and intrusions behaviorally but also by looking at their neural correlates.

Since the pioneer study of Milner (1964), commission of errors has been linked to frontal lobe impairments. The emergence of imaging techniques in the last decade further allowed the identification of frontal pathways associated with verbal production impairments such as the frontal aslant tract (FAT), anterior thalamic radiation (ATR), and uncinate fasciculus (Catani et al., 2013; Cipolotti et al., 2020). Empirical data have shown the importance of these tracts with VF deficits in primary progressive aphasia (Catani et al., 2013). Also, their involvement is confirmed in studies stimulating electrically some of the mentioned tracts (i.e., the FAT) causing speech arrest (Chernoff et al., 2019). Similarly, lesion (e.g., (Kinoshita et al., 2015) and imaging studies (e.g., Sharp et al. (2010) have demonstrated the involvement of frontal pathways in word generation, verbal fluency, stuttering, and inhibitory abilities (for review see: Dick et al., 2019). Based on this evidence, it seems highly probable that commission of errors in semantic VF is related to the integrity of frontal lobe tracts, which are known to degenerate in very early phases of AD, including preclinical stages (Caballero et al., 2018).

Thus, the goal of the present study is two-fold: (a) perform a fine behavioral evaluation of the occurrence of perseverations and intrusions in semantic VF in the category most frequently employed for the assessment of dementia (Moreno-Martinez et al., 2017), namely “animals” (Ardila et al., 2006); and (b) assess frontal tracts purportedly associated to error behavior. Three groups of participants were included in the study: patients with mild AD, healthy age-matched older controls, and healthy younger adults.

To address the first goal of the study, we conducted a first stage where we combined computational automatization for classification of clusters (i.e., latent Dirichlet allocation method; Blei et al., 2003) in order to avoid idiosyncratic decisions together with a time-course analysis of word generation. Then, to address the second goal, we conducted a second stage of the study where we applied diffusion tensor imaging (DTI) techniques to evaluate the white matter integrity of frontal tracts of the participants. In this second stage, we assessed the structural status of frontal tracts as well as their relationship to error commission. For the assessment of the association between error occurrence and frontal tract integrity, only data from participants committing errors were analyzed. We expect that error behavior arises mainly in the patient group and, to some degree, in the older healthy controls. Moreover, we hypothesize that all subjects with high recurrence of errors, disregarding which group they belong to, will show compromised integrity of frontal tracts. However, we do expect that the healthy older controls will be less prone to committing errors, while the patients with AD are expected to show a high incidence of errors. Degree of tracts’ deterioration is expected to be relative to degree of error incidence.

## Materials and Methods

### Participants

An initial sample of seventy-two participants was recruited for the present study, with 24 subjects on each of the following groups: healthy young adults, healthy older adults, and patients with mild AD. Due to technical troubles, we did not have complete speech/spectographic data for one healthy older adult. Moreover, five of the patients turned out to have other etiologies than AD. Thus, the latter six individuals were excluded from the study.

#### Stage 1. Participants Included in the Behavioral Analyses of Errors

A sample of sixty-six Norwegian individuals was retained for the first stage of the study. Of these, 24 were healthy young adults (13 females, 11 males; age: M = 30.2 years, SD = 5.9), 23 were healthy older adults (9 females, 14 males; age: M = 67 years, SD = 8.2), and 19 were patients with mild AD (9 females, 10 males; age: M = 64 years, SD = 10.1). Inclusion of the younger group was deemed convenient in order to evaluate commission of errors and status of frontal tracts of the healthy older group. All the participants were right-handed native Norwegian speakers from North Norway. The patients were recruited at the University Hospital of North-Norway from the Neurology and Geriatrics departments. Only patients with mild AD were enrolled in the study. Following consensus criteria for mild AD (see, e.g., Versijpt et al., 2017), these patients had scores on the Mini Mental State Examination (MMSE) (Folstein et al., 1975) above 20. Each patient underwent standard clinical examinations for the detection of AD, including cerebrospinal fluid (CSF) concentrations of tau, phosphorylated tau, and β-amyloid. Diagnosis was settled by an experienced neurologist or/and geriatrician according to the DSM-IV and NINCDS-ADRDA (Mckhann et al., 1984) criteria for probable AD. Importantly, all the patients were included in the study only upon verification of AD diagnosis after a year of the initial diagnosis.

The healthy older controls were community-dwelling persons recruited through advertisements in a local senior citizen center, flyers, and by means of word of mouth. This group was selected to match as much as possible the patient group for age and gender. Participants in the younger group were university students recruited through advertisements in the university campus. All the participants were tested for cognitive status with the MMSE and a comprehensive neuropsychological assessment. For controls, only participants with an MMSE score ≥ 28 and not depressed according to the adapted criteria (Rodríguez-Aranda, 2003) for the elderly on the Beck Depression Inventory (BDI) (Beck et al., 1988) were included in the study. The healthy controls had no history of psychiatric or neurological illness, tumors, or drug or alcohol abuse. A neuroradiologist screened the MR images for major pathologies such as infarctions or tumors. Involvement in the study was voluntary, and written consent was signed before testing. The healthy groups provided signed informed consent prior to participation in the study. As for the patients, only those individuals retaining the ability to give informed consent at the time of testing were enrolled in the investigation. An initial interview was conducted to obtain demographic information. The study was approved by the Regional Research Ethics Committee.

#### Stage 2. Participants Included in the Structural Assessment of Frontal Tracts and Their Relationship to Error Occurrence

All sixty-six participants included in the first stage of the study had MRI data enabling anatomical comparisons of the selected frontal tracts, which was deemed important to establish the status of tracts in the older groups relative to the younger individuals. Nevertheless, only the older groups were followed-up in this second stage, as the younger individuals committed almost no errors. Thus, the evaluation of the association between integrity of frontal tracts and recurrence of errors was performed exclusively on the healthy older adult group and mild AD group. Both older groups were further subdivided into low-error and high-error subgroups. Description of how we obtained the subdivisions is provided in the later section “Subdivision of Older Groups”. Nine healthy older participants conformed to the control low-error subgroup (Con_low–error_) (M age = 71.3, SD = 3.1; 5 females), while 14 were assigned to the control high-error subgroup (Con_high–error_) (M age = 64.3, SD = 9.3; 4 females). As for the patients, 9 were allocated to the AD low-error subgroup (AD_low–error_) (M age = 62.3, SD = 11.7; 5 females) and 10 to the AD high-error subgroup (AD_high–error_) (M age = 65.6, SD = 8.7; 4 females).

### Procedures Behavioral Analyses

#### Verbal Fluency Assessment Scoring and Classification of Errors

The “animals” category was chosen for the present study, as this is the category of semantic VF most reported in the literature that discerns between normal aging and dementia (Rofes et al., 2020). We evaluated this task in an adapted computerized version developed in our laboratory (for detailed description of the adaptation see Rodríguez-Aranda and Jakobsen, 2011). Shortly, the participants wore a headset with microphone for recording answers while they sat in front of a computer screen. The word “animals” (dyr in Norwegian) was presented *via* the E-prime software (Psychology Software Tools, Pittsburgh, PA, United States), and the participants were asked to start producing words belonging to the category within 1 min as fast as the word appeared on the screen. They were explicitly asked to generate different types of animals, as fast as possible, and not to repeat any exemplar. The word remained present on the screen during the whole trial.

Answers were recorded simultaneously on a computer program (CSL 4500, Kaypentax), on a digital recorder, and manually by the experimenter in charge of the testing. Thereafter, two different coders checked the answers to ensure reliability of the results. Next, the same coders carried out manually the regular scoring of correct answers, which is simply the accounting of total number of correct generated words belonging to the “animals” category. In addition, the coders identified perseverations and intrusions. An intrusion was defined as an answer that did not pertain to the “animals” category, while perseverations were words repeated at any point during the 1-min trial after the first production of the word in question (e.g., “tiger, car, elephant, lion, tiger”; car = 1 intrusion; tiger = 1 perseveration).

#### Identification of Clusters

A regular score in the evaluation of semantic VF is that of clustering (Troyer et al., 1997). During semantic VF performance, subjects produce words matching a given category, for instance, animals. Word generation takes place by producing subclassifications of the required category, for example: farm animals, birds, mammals, or four-legged animals. Thus, word production in semantic VF occurs through subgroups or clusters. According to Rofes et al. (2020), a cluster can be defined as group of words belonging to a semantic family, which is sub-categorized under the superordinate category. The methods employed for identification of clusters has varied, from subjective *ad-hoc* decisions of the human coder to analytic methods based on automatic speech transcription and machine learning classifiers (Montemurro, 2014; Holmlund et al., 2019). In this study, we apply the latent Dirichlet allocation (LDA) technique, which is a Bayesian method for topic extraction in sampling of documents (Blei et al., 2003). LDA is an ameliorate approach of probabilistic latent analysis (pLA), which in turn is an improved technique of latent semantic analysis (LSA) (Anaya, 2011; Rosenstein et al., 2015) widely used in psycholinguistic research (e.g., Pereira et al., 2013).

### Latent Dirichlet Allocation Analysis

Latent Dirichlet allocation (LDA) is an information retrieval technique, which assumes that multiple abstract topics (latent semantic structure) exist in a document, and it extracts them quantitatively by calculating the probability of co-occurrence of words in a document. This type of model ignores the order of words and for this reason, it is called “bag-of-words model” (Blei et al., 2003). According to LDA, a category is the highest concept assumed of a semantic structure (e.g., animals), while a subcategory is a subset in the semantic structure (e.g., sea animals or insects). Thus, LDA identifies through co-occurrence of words in documents specific “topics,” which correspond to the concept of a “subcategory.” Once identification of topics is performed by LDA, clusters can be defined in a sequence of VF responses. It is, therefore, important to note that “cluster” and “topic” cannot be interchangeably used.

Human evaluators rely on idiosyncratic beliefs to classify a word as pertaining or not to the animal category based on what a specific person knows about “animals.” However, because LDA is not based on such notions, it enables us to appraise whether wrong answers are actually semantically or lexically related to the generated topics. The topic identification based on LDA likely corresponds to the classification by animal subcategories but not necessarily corresponds to them in the same way that human coders would appraise.

#### Estimation of Topic Probabilities and Clusters by Latent Dirichlet Allocation Analysis

In the present study, we used 180 unique words in LDA analysis that were produced by our participants. We intentionally included wrong words (i.e., intrusions; e.g., rose) to assess semantic associations *vis-à-vis* “error” responses. We used the Norwegian version of the Wikipedia database (nowiki-20181020-pages-articles.xml.bz2, 495,898 articles) as a dataset. However, we reduced the document dataset to 80,405 articles containing only the response words. In this regard, the use of LDA is advantageous, as this technique creates a generalizable model to unknown data that suits the relatively limited number of articles available in Norwegian. According to the method of Blei et al. (2003), we conducted LDA analyses on the data using Rstan with the following parameters: number of topics = 3-15 and α = 0.2, 0.3, 0.4, 0.5, and 1. Thus, we obtained 65 possible models (3 × 15 = 65), and then we selected a model with the number of topics = 14 and α = 0.3 based on leave-one-out cross-validation (LOOCV) to define the model. Based on the selected model, we calculated topic probability, which is the probability of the existence of a latent topic when a specific word appears in a document. One word has multiple topic probabilities. In the present model, 14 topic probabilities were obtained, which connected to 14 possible subcategories. The sum of the topic probabilities for a word is 1 (100%). The word list and topic probabilities of the model used in the study are provided as Supplementary Material (see Supplementary Data File 1).

#### Definition of Clusters by LDA Analysis and Error Identification

In this study, a cluster in VF responses was defined based on topic probability; when topic probabilities to the same topic are higher than the criteria (= 1/14 × 0.5) in two consecutive word responses. The criterium of two consecutive responses was adopted in agreement with suggested norms by Troyer et al. (1997) and Rich et al. (1999). After identification of the clusters by LDA, we were able to locate the errors in the timeline of execution extracted from the time-course analysis. In addition, we counted the number of responses within a cluster, that is, the number of responses constituting a cluster. Note that it is possible that multiple topic probabilities exceed the clustering criteria within one cluster because of the definition of topic probability. In other words, an ongoing topic can overlap and transpose to another topic within a cluster. As an example, we present the following sequence: “monkey (topic 3), gorilla (topic 3) chimpanzee (topic 3 and 9), kangaroo (topics 8 and 9).” This group of words is considered a cluster, while the transposition from topics 3 to 9 occurs at “chimpanzee.”

### Time-Course Analyses

The deployment in time of all answers, including perseverations and intrusions, was analyzed with a speech lab system (CSL 4500, Kaypentax). In this analysis, the acoustic signal is visually and auditorily examined to settle the exact time of occurrence of each intrusion and perseveration all along the 1-min trial of the execution.

Two types of time-course analyses were conducted. First, we applied a strategy widely used in the literature (e.g., Rosen, 1980; Crowe, 1998; Kim et al., 2011), consisting of partitioning the VF trial into15-s phases to analyze performance by time period. We, thus, quantified the total number of responses (i.e., correct responses + errors) and types of errors (i.e., intrusions vs. perseverations) separately by phase and by group. The rationale was to obtain patterns of performance in overall word production and most importantly in error production as a function of time for each specific group. Previous research (e.g., Rohrer et al., 1999) focusing only on the time course of correct word production might have led to incomplete or wrong conclusions, as VF performance was only partially analyzed, that is, the occurrence of errors was not included. This selective way of analyzing VF might have prevented us from delineating important aspects of the processing speed issue in aging and dementia.

Keeping this line of reasoning, we conducted a second strategy where we calculated in-between intervals of errors. This procedure enabled us to test whether the incidence of errors had a relationship to the time used to produce the inaccuracies. This second approach aims, from a different perspective, to assess the role of processing speed in error occurrence. To achieve this goal, complementary information related to the number of correct words generated between errors was needed. Therefore, we quantified the total number of words produced amid error occurrences. If correct words are produced in between errors at a similar rate across the trial in all the groups, it will discard slowing of processing speed as a central factor of group differences. Thus, calculation of in-between error intervals was restricted to what we considered the best three alternatives presenting comprehensible information: (a) between same perseveration (i.e., same repeated word); (b) between intrusions; and (c) between errors of any type (i.e., “intrusion-intrusion,” “intrusion-perseveration,” “perseveration-perseveration,” and “perseveration-intrusion”). For calculations of (b) and (c), we excluded data with only one error.

### Statistical Analyses for Behavioral Data

One-way between-subject ANOVAs were conducted to show group differences in demographic variables and in the number of responses, perseverations, and intrusions. According to initial sample size calculations for a three-level one-way ANOVA, we needed 24 individuals in each group to attain 85% statistical power and reach large effect sizes (*f* = 0.4) at a significance level of 0.05. Significant interactions or main effects involving group differences were followed up with appropriate *post-hoc* analyses. Chi-square tests were performed to detect possible differences in error production among the groups, and to detect differences in the time course between the two types of errors. Furthermore, correlation coefficients were calculated to quantify the relationship among the number of responses, errors, and topics.

### Procedure DTI of Frontal Tracts and Their Association to Errors

#### MRI Acquisition

The participants were scanned in a 1.5T Phillips Intera MR scanner. Diffusion-weighted images were obtained using a single-shot SE-EPI sequence with TE/TR = 79/11,663 ms, SENSE acceleration factor 2, FOV 252 X 252 mm, and in-plane resolution 2.25 X 2.25 mm^2^ in 70 axial slices (slice thickness of 2.25 mm). Diffusion gradients were applied in 15 directions with b = 1,000 s/mm^2^, and a volume without diffusion weighting was acquired. Two common DTI metrics were assessed: fractional anisotropy (FA), which denotes the strength of diffusion directionality, and mean diffusivity MD, which indicates the overall rate of diffusivity (Madden and Parks, 2017). In aging, a decrement in FA has been reported, which often is coupled with an increment in MD (de Groot et al., 2016). These events suggest degeneration in white matter in terms of tissue loss and replacement of the damaged tissue by free water (Pfefferbaum and Sullivan, 2003). Of interest for the present study is that the magnitude of FA and MD changes is reported to be greater in AD than in normal aging (Caballero et al., 2018).

#### DTI Preprocessing

Preprocessing and statistical analysis of the DTI data were performed using the FSL software library (v5.0.9). The diffusion-weighted images were corrected for motion and eddy currents using FLIRT (Jenkinson and Smith, 2001). A brain mask was created per participant using BET (Smith, 2002). Diffusion tensor, fractional anisotropy (FA), and mean diffusivity (MD) were calculated using the DTIFIT tool of FMRIB’s Diffusion Toolbox of FSL.

##### Anatomical Comparison of Frontal Tracts

Anatomical comparisons of the three initial groups were deemed appropriate to understand the status of frontal tracts of the older groups relative to the younger adults. In this way, we could appreciate the degree of tract deterioration in the patients with AD relative to the older control, and in the healthy older adults relative to the younger participants. Thereafter, we proceeded to evaluate the relationship between frontal tract integrity and error commission in four subgroups of the older participants (mild AD and healthy older controls).

##### Evaluation of Frontal Tract Status and Their Relationship to Errors in the Older Groups

The younger adult group committed practically no errors, and for this reason, this group was excluded in this part of the analyses. An overview and explanation of the reasons for exclusion can be found in Supplementary Material. The older groups were then subdivided into low-error and high-error subgroups.

#### Subdivisions of Older Groups

Because of scarce availability of error data, we subdivided the patients and older controls relying on the total score of errors, that is, on the sum of both intrusions and perseverations. The reasons for this decision are methodical and theoretical. To begin with, it is not reasonable to consider a division of groups based on type of errors because of the low number of occurrence by error type. As for the theoretical standpoint, the literature suggests that both intrusions (e.g., Desgranges et al., 2002) and perseverations (e.g., Corrivetti et al., 2019) are related to frontal impairments. Thus, it seems logical to evaluate the integrity of frontal tracts in relation to general error production. Subgrouping of the older and AD groups was based on a cut-off point using the median of total errors (intrusions + perseverations) from each group. The median values employed were 0.5 for the older adults and 1 for the patients with AD. Thus, any participant showing a score equal or above the respective value for his/her group was assigned to the “high-error subgroup,” while those having a score below the median value were assigned to the “low-error subgroup.”

### Statistical Analysis for Imaging Data

Voxelwise statistical analyses of the FA and MD data were carried out using TBSS (Tract-Based Spatial Statistics, version 1.2; Smith et al., 2006), part of FSL (Smith et al., 2004). Two sets of analyses were performed. For the first set, an anatomical evaluation of the tracts was conducted across groups without subdivisions, and the younger group was included. Hence, the younger group, the healthy older controls, and patients with AD were compared. The reason to include the younger group in this initial stage was for evaluation of the integrity of the tracts of the older controls. Thus, FA and MD data of the three groups were aligned into the FMRIB58_FA standard space using the nonlinear registration tool FNIRT (Andersson et al., 2007; Andersson et al., 2007). The comparisons were performed along the three selected frontal tracts: (1) anterior thalamic radiation (ATR), (2) frontal aslant tract (FAT), and (3) uncinate fasciculus (UNC).

For the second set of analyses, comparisons between the four subgroups of older controls and patients were performed. Again, we evaluated the selected tracts FAT, ATR, and UNC. For these comparisons, FA and MD data were aligned to the most representative image of the sample, because all the images corresponded to seniors. In this second set of analyses, we conducted two DTI solutions. The first solution was conducted voxel-wise within each tract and separated by hemisphere. Voxel-wise statistics were performed with the Randomize tool (v2.5; part of FSL), a permutation-based method (Winkler et al., 2014). We used 5,000 iterations, a threshold-free cluster enhancement for multiple comparison correction and a significance threshold of *p* < 0.05 for all the statistics. Age and sex were included as confounders in all the analyses. Since there were no differences in education between the healthy older adults and patients with AD, this variable was not entered as a confounder. In the second solution, we performed a global assessment of complete tracts by multivariate analysis using the SPSS software (version 24) to test interaction effects on the mean values of FA and MD across all the voxels of each tract. In this way, possible interactions by group, sex, age, tract, and hemisphere were tested. Because of multiple comparisons, the Sidak correction was applied.

## Results

### Behavioral Analyses

#### Demographics

Demographic characteristics of the groups are shown in Table 1. Since some of the initial participants were excluded from the study, we calculated the statistical power of our remaining sample. Although this calculation of unbalanced ANOVA is not straightforward, the sample sizes in the one-way three-level analysis (*n* = 19, 23, and 24) were large enough to detect large effect sizes (*f* > 0.4) with a statistical power of = 0.8. Hence, significant differences among the groups were found for MMSE (*F* (2, 63) = 26.5, *p <* 0.001, η_*p*_^2^ = 0.45) in which the patients with AD presented considerably lower scores than the healthier groups. It is noteworthy that the older controls had a very similar score on MMSE than the younger adults. Years of education showed significant group differences (*F* (2, 63) = 19, *p <* 0.001, η_*p*_^2^ = 0.38), as both the older groups had less formal schooling than the younger participants. Evidently there were significant group differences in age (*F* (2, 63) = 148.2, *p <* 0.001, η_*p*_^2^ = 0.82), but not in sex (χ^2^ (2) = 1.1, *p* = 0.56, NS). Regarding age and years of education, multiple comparisons showed significant differences between the young and the two older groups for education (*t*(63) = 5.65; *t*(63) = 4.95; *p* < 0.05), whereas the healthy older and AD groups did not differ significantly in age (*t*(63) = 2.99, *p* = 0.24) or education (*t*(63) = 1.04, *p* = 0.3).

**TABLE 1:** Demographics, MMSE scores by group.**^a^**

	Young group (*n* = 24)	Healthy older group (*n* = 23)	Mild AD Patients (*n* = 19)	*F*(2, 63)	*p*-value
Female	13 (54.16%)^a^	9 (39.13%)	9 (47.36%)		
Age (years)	30.2 (5.9)	67.0 (8.2)	64.0 (10.1)	148.2	**0.001**
Education	17.1 (2.3)	12.1 (3.7)	11.0 (3.5)	19.0	**0.001**
MMSE	28.9 (0.9)	28.7 (0.7)	25.0 (3.4)	26.5	**0.001**

*NB: MMSE = Mini-Mental State Examination. ^a^Mean (SD) or N (%). Bold values indicate that they are statistically significant (p < 0.05).*

#### Standard Scores for Semantic Verbal Fluency

Table 2 presents standard results for the “animals” category in terms of mean values for total number of correct responses, intrusions, and errors generated during the whole minute by group. The results showed significant group differences in correct number of answers (*F* (2,63) = 16.06, *p <* 0.001, η_*p*_^2^ = 0.34) and intrusions (*F* (2,63) = 5.43, *p <* 0.01, η_*p*_^2^ = 0.14). Group contrasts for perseverations did not yield significant differences (*F* (2,63) = 2.22, *p* = 0.11, NS, η_*p*_^2^ = 0.07). The multiple comparisons with Holm’s method showed significant group differences for correct answers between the patient group and both healthy groups (*p <* 0.05). The same *post hoc* analysis revealed that group differences for intrusions were significant between the patient group and both healthy groups (*p <* 0.05).

**TABLE 2:** Means and SD for standard scores of the “animal” category in the 1-min trial.^a^

	Young group (*n* = 24)	Healthy older group (*n* = 23)	Mild AD Patients (*n* = 19)	*F* (2, 63)^a^	*p*-value
Total number correct answers	20.6 (1.0)	18.8 (1.4)	12.9 (0.97)	14.9	**0.001**
Total number of intrusions	0.00 (0.00)	0.09 (0.28)	0.95 (1.87)	3.8	**0.01**
Total number of perseverations	0.46 (0.66)	0.87 (1.49)	1.26 (1.59)	3.0	0.11

*M = Mean, SD = standard deviation. Significant values are presented in bold. ^a^Mean (SD).*

### LDA Analysis

#### Cluster Identification

Using the LDA technique, fourteen topics were extracted. Thereafter, the clusters were defined in a sequence of VF responses based on topic probability. A summed topic probability is the aggregated value of topic probabilities of a set of responses pertaining to a topic. In the following section, we present summed topic probabilities exclusive to errors. The corresponding results of summed topic probabilities to correct responses can be found in Supplementary Material I, which help understand the distribution of topics by type of generated word (correct vs. errors).

#### Error Production by Topic Probability

**FIGURE 1:**
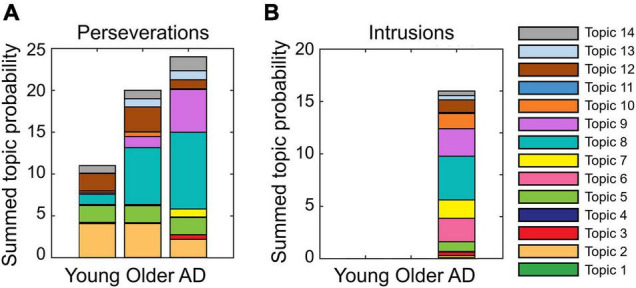
Proportion of summed topic probabilities for each topic in **(A)** perseverations and **(B)** intrusions. Note that the intrusions committed by healthy older adults, (“kemse” and “lojør”) are unknown utterances. As such, they are not found in Wikipedia articles, and accordingly, they have no topic probabilities. Likewise, two out of the 18 intrusions committed by patients with Alzheimer’s disease (AD) (“rose bed” and “pusi”) were not found in the Norwegian Wikipedia articles.

Perseverations: patterns of summed topic probabilities differ across groups (Figure 1A). This is true for topic 2 in the young adults, for topics 2 and 8 in the older controls, and topics 8 and 9 in the patients with AD. From the analyses of cluster identification, it became evident that more than half of the perseverations were produced within a cluster, which means 64% in the young group, 55% in the older adults, and 62% in patients with AD.

Intrusions: Figure 1B shows that the patient group mostly committed intrusions with the highest summed topic probability in topic 8. About half of the intrusions (56%) were found within clusters in the AD group. These results showed as a whole that more errors of any type were produced in topics with more words (to appraise this statement, refer to Supplementary Material 1).

#### Relationship Between Clusters and Errors in Topic Probability

**FIGURE 2:**
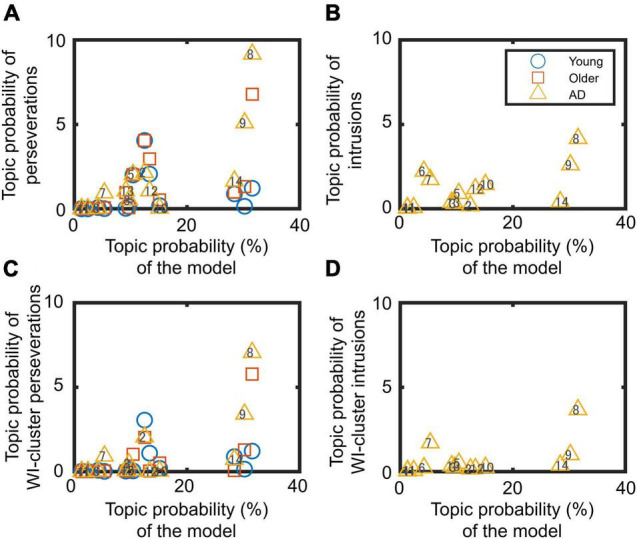
Relationship between summed topic probabilities of the topic model and errors: **(A,C)** perseverations and **(B,D)** intrusions. Panels **(C,D**) concern within-cluster (WI-cluster) errors. Each marker indicates the data for one topic (from 1 to 14 topics).

Figure 2 shows the relationship between the summed topic probabilities for the topic model and the two types of errors (perseverations and intrusions). These analyses are of importance, as they allow us to assess the degree of association between errors and the 14 generated topics. The correlation coefficients between the summed topic probabilities for perseverations and topic model on each topic were *r* = 0.22, 0.60, and 0.79 in the younger, older, and AD groups, respectively (Figure 2A). The correlation coefficients (*r*) between the summed topic probabilities for intrusions and the topic model in each topic for the patients was 0.60 (Figure 2B). Similarly, the positive relationship between the errors and the topic model in the summed topic probabilities are also observed in within-cluster errors (Figures 2C,D). The reader should note that in the intrusion panels (Figures 2B,D) only the summed topic probabilities for the AD group are presented, because the healthy groups never or scarcely produced intrusion errors. Some of the intrusions committed by the healthy older adults, (“kemse” and “lojør”) and AD group (“rosebed” and “pusi”) were not factual words; hence, they were not included in any topic probability.

### Time-Course Analyses

#### Generation of Total Number of Responses by 15-s Phase, by Group

The illustration on the deployment in time of total number of generated words including errors across the 4 phases is presented in Supplementary Material (Supplementary Figure 2). The results showed that word production decreased as time passed in all the groups. A two-factor ANOVA revealed a significant main effect of group (*F* (2, 63) = 9.91, *p <* 0.001, η_*p*_^2^ = 0.24) and phase (*F* (3, 189) = 55.9, *p <* 0.001, η_*p*_^2^ = 0.47), but the interaction effect was not significant (*F* (6, 189) = 2.08, *p* = 0.057, η_*p*_^2^ = 0.06). *Post hoc* calculations revealed that the number of responses of the AD group was significantly lower than that of the young and older groups (*t* (63) = 4.31; *t* (63) = 3.29) but did not differ between the latter two groups (*t* (63) = 1.03). The analysis also showed significant differences among all the phases (*t* (63) = 4.4 for phases 1 and 2*; t* (63) = 4.86 for phases 2 and 3; *t* (63) = 2.97 for phases 3 and 4; *t* (63) = 9.41 for phases 1 and 3; *t* (63) = 11.68 for phases 1 and 4; *t* (63) = 4.86 for phases 2 and 3; *p <* 0.05).

#### Perseverations and Intrusions by 15-s Phase, by Group

**FIGURE 3:**
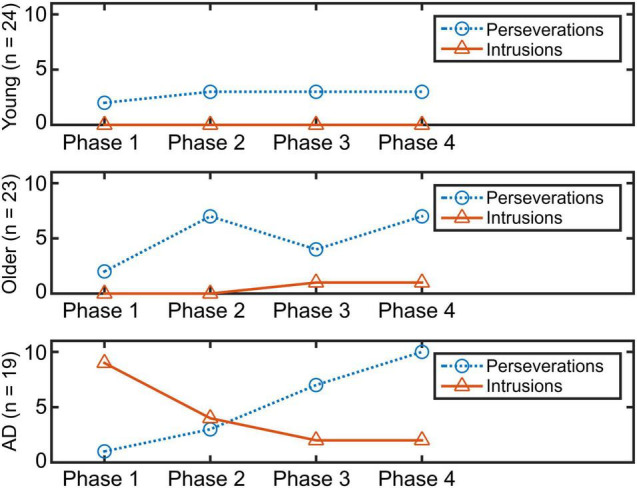
Time course of each error in four phases.

Figure 3 shows the time course of the total number of errors in the four phases. To detect differences in the time course of two types of errors, chi-square tests were conducted. Whereas the tests did not find any statistical differences between the total number of errors and phases in younger and older groups (χ^2^(3) = NA, *p* = NA, *w* = NA; χ^2^(3) = 1.73, *p* = 0.63, *w* = 0.28), a significant difference in the time course among the errors in the AD group (χ^2^(3) = 14.4, *p* = 0.002, *w* = 0.61) was found. A residual analysis revealed significant differences in phases 1 and 4 (*p <* 0.05).

#### In-Between Intervals of Errors

In-between intervals of perseverations: this calculation was performed by measuring the time interval between a generated word and the repetition of the same word (e.g., time between “lion and lion” or “fish and fish”). For these data, we considered 9 younger adults (M = 24.2 s, SD = 16.88), 12 healthy older adults (M = 21.64 s, SD = 13.69), and 10 patients with mild AD (M = 22.81 s, SD = 8.09). A one-way ANOVA showed no significant group differences (*F* (2, 28) = 0.09; *p* = 0.91, NS, η_*p*_^2^ = 0.01).

In-between intervals of intrusions: this measurement was not carried out because of restricted amount of data. None of the younger participants committed intrusions, while only 2 older adults committed 2 intrusions. From the patient group, there were 18 intrusions, and only 2 participants had enough data to compute the in-between interval calculation.

In-between intervals of any error type: for these data, 9 younger adults (M = 31.11 s, SD = 16.56), 14 healthy older adults (M = 33.86 s, SD = 13.74), and 15 patients with mild AD (M = 31.56 s, SD = 13.55) were available. A one-way ANOVA showed no significant group differences (*F* (2, 35) = 0.13; *p* = 0.88, NS, η_*p*_^2^ = 0.01).

These analyses consistently showed that there were no practical differences in error intervals among the groups. Note that the statistical power in these analyses is low because of limited sample sizes in each group. Therefore, effect sizes are particularly important.

#### Word Production Within Intervals of Error Production

Because of availability of data, only the total number of produced words during in-between intervals of perseverations and of any error type was possible to analyze. The results concerning perseverations demonstrated that the 9 younger adults (M = 7.1 words, SD = 5.4), 12 healthy older adults (M = 7.6 words, SD = 3.5), and 10 patients with mild AD (M = 6.1 words, SD = 3.2) generated a similar number of responses. A one-way ANOVA showed no significant group differences (*F* (2, 28) = 0.38; *p* = 0.68, NS, η_*p*_^2^ = 0.03). As for the number of words in between intervals of any error type, we obtained limited data. Only 2 younger adults (M = 4.5 words, SD = 0.71) and 3 healthy older adults (M = 4.56 words, SD = 5.6) had useful outcomes for this analysis, while there were data available from the 10 patients with mild AD (M = 2.43 words, SD = 3.23). The corresponding one-way ANOVA showed no significant group differences (*F* (2, 12) = 0.56, *p* = 0.58, NS, η_*p*_^2^ = 0.09). Again, due to low statistical power in these analyses, effect sizes are of relevance.

### DTI Analyses

#### Anatomical Comparisons of Frontal Tracts Among Young, Older, and Mild AD Groups

The analyses of FA and MD values in the frontal tracts comparing the young adults, healthy controls, and patients with AD are presented in Supplementary Table 1. The young adults showed increased FA compared to the healthy seniors and patients with AD, separately, in bilateral ATR and FAT, and in left UNC. The healthy seniors showed larger FA than the patients with AD only in the left ATR. For MD, the healthy seniors compared to the young adults, showed increase in the right FAT. The patients with AD presented increased MD in right ATR and left FAT compared to the young adults. Compared to the healthy seniors, the patients with AD showed increased MD in bilateral FAT, and left ATR and UNC. For all the comparisons, *p*-level was set at 0.05.

#### Assessment of the Integrity of Frontal Tracts and Errors in Older Subgroups

In this section, we will present VF results pertaining to the fours subgroups from the older groups. Complete data of error commission ratio of the original three groups (young, healthy older, and patients with mild AD) can be found in Supplementary Material II and Supplementary Figure 3.

Error scores across the four subgroups were as follows: Con_low–error_, M = 0, SD = 0; Con_high–error_, M = 0.8, SD = 0.8; AD_low–error_, M = 0.3, SD = 0.3; and AD_high–error_, M = 1.85, SD = 0.8. A chi-squared test conducted on the four subgroups of older controls and patients showed no group differences for sex (χ^2^ (3) = 2.38, *p* = 0.5, NS). Similarly, a one-way ANOVA showed no significant differences for age (*F* (3, 38) = 1.76, *p* = 0.17, NS) or education (*F* (3, 38) = 1.3, *p* = 0.29, NS).

**FIGURE 4:**
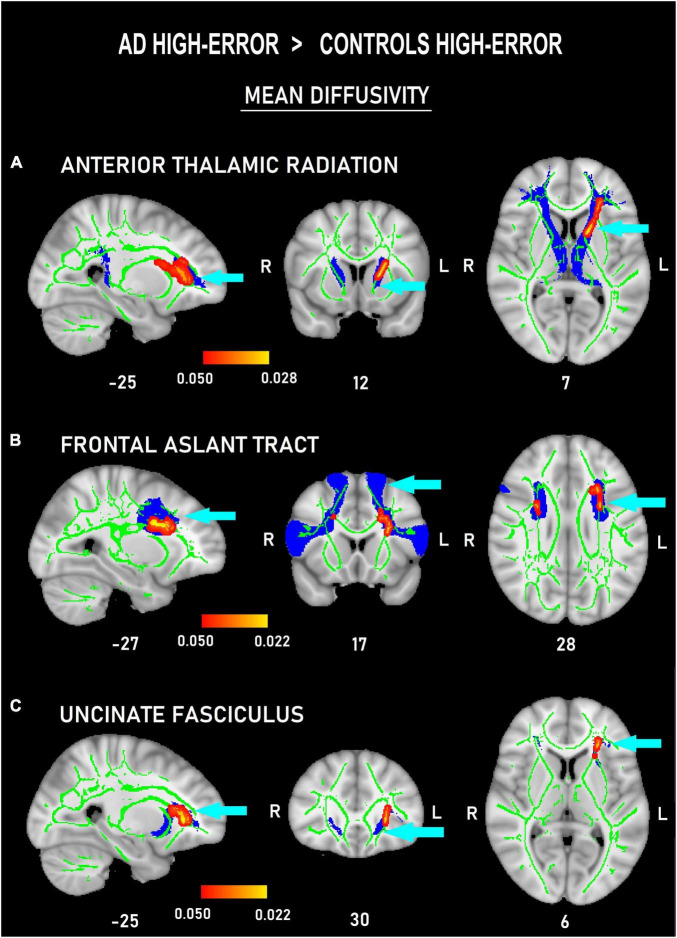
Probability maps of tract-based spatial statistics showing clusters with increased mean diffusivity in patients with AD committing a high number of errors versus controls committing a high number of errors in three frontal white-matter tracts: anterior thalamic radiation **(A)**, frontal aslant tract **(B)**, and uncinate **(C)** fasciculus. Sagittal **(left)**, coronal **(middle)**, and axial **(right)** radiological views with corresponding MNI coordinates. Red-yellow shade bars indicate the significant *p* value ranges for every tract. The fractional anisotropy skeleton is shown in green. The area corresponding to each tract is shown in blue.

**FIGURE 5:**
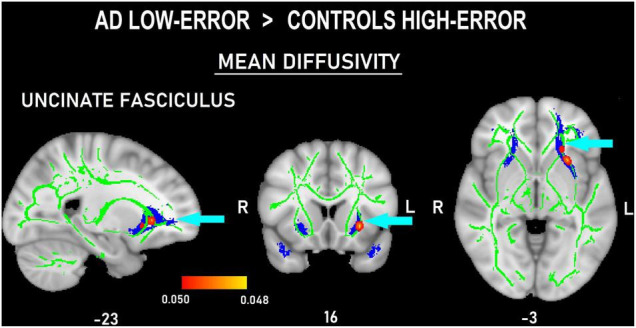
Probability map of tract-based spatial statistics in the uncinate fasciculus showing clusters with increased mean diffusivity in patients with AD committing a low number of errors compared to controls committing a high number of errors. Sagittal **(left)**, coronal **(middle)**, and axial **(right)** radiological views with corresponding MNI coordinates. The red-yellow shade bar indicates the significant *p* value range. The fractional anisotropy skeleton is shown in green. The area corresponding to the uncinate fasciculus is shown in blue.

The voxel-wise comparisons among the four subgroups only showed differences in MD measures of the frontal tracts. In the comparisons between the high-error groups, AD_high–error_ showed increased MD values in left ATR (Figure 4A) and UNC (Figure 4C), and bilateral FAT (Figure 4B) compared to Con_high–error_ (*p <* 0.05, *d* = 2.34). Moreover, AD_low–error_ also showed marginally larger MD measures than Con_high–error_ in the left UNC (*p <* 0.05, *d* = 2.42; Figure 5).

The results from the multivariate analysis on FA and MD measures per hemisphere and tract by subgroups (second solution) are shown in Table 3. These data revealed main effects of age (*p* = 0.041, η_*p*_^2^ = 0.166), group (*p* = 0.005, η_*p*_^2^ = 0.235), and tract (*p* = 0.001, η_*p*_^2^ = 0.4), but no main effect of hemisphere (*p* = 0.99) or sex (*p* = 0.51). Specifically, the three main effects were present in MD measures (*p* = 0.022, η_*p*_^2^ = 0.138; *p* = 0.017, η_*p*_^2^ = 0.242; and *p* = 0.003, η_*p*_^2^ = 0.196, respectively; see bottom part of Table 3).

**TABLE 3:** Comparisons for the integrity of three frontal tracts between the subgroups of older controls and patients.

		Con_low–error_ (n = 9)		Con_high–error_ (n = 14)		AD_low–error_ (n = 9)		AD_high–error_ (n = 10)		MANCOVA	Interactions	GroupContrasts
								
		*M*	*SD*	*M*	*SD*	*M*	*SD*	*M*	*SD*	Age / Group / Tract	Tract*Hemisphere / Hemisphere*Sex	1vs4 / 3vs4
**Fractional Anisotropy**												
*Anterior Thalamic*	R	0.3952	0.0209	0.4047	0.0199	0.3962	0.0211	0.3962	0.0205	ns / ns / ns	ns / ns	ns / ns
*Radiation*	L	0.3991	0.0176	0.4031	0.0164	0.3949	0.0252	0.3899	0.0237			
*Frontal Aslant Tract*	R	0.3623	0.0249	0.3756	0.0210	0.3616	0.0206	0.3678	0.0226			
	L	0.3780	0.0184	0.3831	0.0216	0.3685	0.0197	0.3790	0.0199			
*Uncinate Tract*	R	0.3769	0.0396	0.3884	0.0233	0.3733	0.0273	0.3829	0.0260			
	L	0.3857	0.0284	0.3935	0.0264	0.3758	0.0323	0.3853	0.0287			
**Mean Diffusivity**												
*Anterior Thalamic*	R	0.0009	0.0001	0.0009	0.0001	0.0009	0.0001	0.0008	0.0000	0.022 / 0.017 / 0.003	0.013 / 0.014	0.041 / 0.040
*Radiation*	L	0.0009	0.0001	0.0009	0.0001	0.0009	0.0001	0.0009	0.0001			
*Frontal Aslant Tract*	R	0.0008	0.0000	0.0008	0.0000	0.0009	0.0001	0.0008	0.0000			
	L	0.0008	0.0000	0.0008	0.0000	0.0008	0.0000	0.0008	0.0000			
*Uncinate Tract*	R	0.0009	0.0000	0.0009	0.0000	0.0009	0.0001	0.0008	0.0000			
	L	0.0008	0.0000	0.0008	0.0000	0.0008	0.0001	0.0008	0.0000			
							Main effects Multivariate Analysis			0.041 / 0.005 / 0.001	0.018 / 0.032	

*Con_low–error_ = control low-error subgroup; Con_high–error_ = control high-error subgroup; AD_low–error_ = AD low-error subgroup; AD_high–error_ = AD high-error subgroup. For the multivariate analysis, only significant comparisons and interactions are presented, p < 0.05.*

Also, there was a significant tract*hemisphere interaction (*p* = 0.018, η_*p*_^2^ = 0.081), indicating that the occurrence of differences in integrity between hemispheres depended on the tract analyzed. The right showed better integrity than the left hemisphere, with specific differences in MD of the FAT (*p* = 0.013, η_*p*_^2^ = 0.137). There was also a significant hemisphere*sex interaction (*p* = 0.032, η_*p*_^2^ = 0.179), where differences between hemispheres were more pronounced in men. Also, this interaction specifically occurred in MD of the FAT (*p* = 0.014, η_*p*_^2^ = 0.157). *Post hoc* pair-wise comparisons showed that group differences only existed in MD of the FAT between AD_high–error_ and both Con_low–error_ (*p* = 0.041) and AD_low–error_ (*p* = 0.04).

Otherwise, no effects for the interaction tract*group (*p* = 0.066), tract*age (*p* = 0.21), tract*sex (*p* = 0.231), group*hemisphere (*p* = 0.482), hemisphere*age (*p* = 0.637), tract*group*hemisphere (*p* = 0.895), and tract*hemisphere*age (*p* = 0.438) were found.

## Discussion

### Behavioral Analyses

The results from the standard scores for animal VF agree with earlier reports (Crowe, 1998; Demetriou and Holtzer, 2017), in which younger participants outperform healthy older adults and patients with AD with regard to correct number of words and considerably fewer errors. In turn, the healthy older group committed more errors than the younger adults, especially perseverations, and generated less number of correct answers. However, as expected, the older adults outperformed the AD group who showed significant decline in word production and higher number of errors in both types. Of importance is that group comparisons for type of error showed that only number of intrusions was significantly different among the groups. As for the time-course analysis, it was observed that word production declined progressively along the 1-min trial across all the groups in a very similar way, but at lower rates for the older adults relative to the younger ones and even lower levels for the patients relative to the healthy elders.

Furthermore, the application of LDA showed that the pattern of the production of topics did not differ among the groups. However, there were specific topics that were more recurrently produced in the older groups, and some of them presented higher incidence of errors. In other words, mostly, frequent topics contained high occurrence of errors.

According to the literature, word production related to large semantic categories, such as “animals” poses difficulties for working memory load and causes higher perseveration rates (Azuma, 2004). Our data revealed that most perseverations (60%) happened within clusters, and a possible explanation for this finding is that generation of errors was triggered by strong lexical connections evoked by specific topics. This occurs in both the healthy subjects and patients with AD. Indeed, different studies have suggested that high frequent words are more prone to induce the appearance of perseverations (Miozzo et al., 2013), and that the animal category accounts among the most frequent categories for the evaluation of semantic VF (Ardila et al., 2006).

It is suggested that most perseverations are caused by limitations in working memory capacity and self-monitoring (Rosen and Engle, 1997). Hence, perseverative responses are more frequent in older adults (Ramage et al., 1999) and in populations suffering from memory and executive disorders such as patients with AD (Azuma, 2004). The present data mainly confirm all the above assertions. As for the intrusions, we found that even though some of the older adults committed few intrusions, the patient group generated primarily this type of error. Again, the LDA analysis showed that almost 60% of the intrusions were found within the clusters. This finding provides support to the idea that even if the deficits in semantic knowledge are present in mild AD, some semantic associations are still preserved (Paganelli et al., 2003). For this reason, production of wrong answers is detected by LDA analyses as conceptually related.

Regarding results from to the time-course analyses, we found a unique pattern of error generation in the patients with AD. While the younger and healthy older adults produced perseverations at a similar rate all along the trial, the pattern of error generation of the patients clearly demonstrated higher incidence of perseverations at the end of the trial with high incidence of intrusions at the beginning of the execution. Whereas it is reasonable that the number of perseverations increases along the trial because of accentuated attentional deficits in the patient group (Miozzo et al., 2013), the high generation of intrusions in the initial stage is more conspicuous. Increased intrusions in AD have been reported in a variety of tasks (Loewenstein et al., 1991; Doubleday et al., 2002). However, to the best of our knowledge, there is no report analyzing the time occurrence of this pathological feature during verbal recall. Earlier studies proposed that commission of intrusions in AD occur because of retrieval difficulties (Desgranges et al., 2002) and inability to suppress inappropriate answers (Shindler et al., 1984). In Shindler et al. (1984) proposed a four-stage process for the occurrence of intrusions. Our study further demonstrates that the chain of events described by Shindler et al. (1984) occurs in the initial stage of word generation in subjects with mild AD.

By assembling the findings from the time-course and LDA analyses, it appears that when the AD group intends to retrieve words, a defective strategic search is launched, which produces semantically related errors (i.e., intrusions) at the same time that it intertwines with the highest possible production of correct words. According to the literature, even if semantic knowledge is degraded in the early stages of AD, some degree of lexical information is still preserved (Barbarotto et al., 1998); therefore, correct answers and semantically related errors appear. It is noteworthy mentioning that only through LDA analyses we can recognize that a great proportion of intrusions are conceptually associated to an animal subcategory. Identification of intrusions based on human coding will not be able to make this link. Thus, the fine analysis of errors by phases demonstrated that the percentage of intrusions relative to total words generated in phase 1 was 9.5% (0.47 intrusions/5 responses on average). Although the proportion is numerically low, it is a real burst of incorrect answers due to a defective lexical search in a degraded semantic system. Remarkably, this event only takes place during the first 15 s. This phenomenon of correct word and intrusion generation relies not only on defective retrieval of semantic information but also on loss of insight in the selection of responses proper to the early stages of AD (Moreaud et al., 2001; Paganelli et al., 2003). From phase 2, other impaired mechanisms take place in the patients with AD, where a more mixed outcome consisting of correct answers, intrusions, and perseverations appears. At this point, the propensity of perseverations begins to increase and reaches its highest levels in phase 4 where 31.3% of their total answers become perseverations (0.53 perseverations/1.68 responses on average). Thus, in the middle of the trial, a shift in activation of impaired mechanisms occurs where perseverations take over.

Now, we wish to draw attention to the findings from group comparisons on the in-between perseveration intervals. In the past, some authors have proposed that semantic memory impairments in mild AD during VF are related to retrieval slowing deficit (Rohrer et al., 1999; Nutter-Upham et al., 2008). Nevertheless, those studies were based on the number of correct generated responses without considering the errors. Thus, our analyses of the in-between error intervals showed no group differences in the in-between lags to generate errors, and the number of responses in these time windows were practically equal across groups, which gives no support to a retrieval slowing in AD.

### DTI Findings and Assessment of the Relationship Between Frontal Tracts and Error Occurrence

The anatomical results demonstrated, as expected, that the younger adults had better white matter integrity than the healthy older controls in all the three tracts. As for the anatomical differences between the healthy controls and patients, only MD values bilaterally in FAT and in left ATR and left UNC were significantly higher in the patients. These data are worth noting, since an earlier comparison of whole brain white matter of these two specific samples did not show significant differences (Rodríguez-Aranda et al., 2016). In that study, only one single DTI measure, namely, the mode of anisotropy (MO) differentiated the groups. However, in the present study, by focusing on particular white matter tracts, mostly from the left hemisphere, we were able to observe anatomical group differences.

In addition, contrasts on the subclassifications of patients and controls demonstrated that the patients had more degenerated tracts than the healthy older adults who committed a high number of errors. Predominantly, these differences were on the left side, even though a small portion of the right FAT also differentiated the subgroups. First, our data showed that the patients with AD with high incidence of errors presented higher MD values in all the tracts. In accordance, a recent investigation (Chen et al., 2020) suggests higher vulnerability of white matter microstructure in the left hemisphere in individuals developing AD. Our findings agree with the suggestion of Chen et al. that white matter deterioration in the left hemisphere is an indication of early signs of the disease.

Furthermore, our results are in line with findings pointing to the association between degeneration of frontal pathways and verbal deficits in different older populations (Papagno, 2011; Kljajevic et al., 2016; Di Tella et al., 2020). More specifically, the fact that left ATR and left UNC were more deteriorated in the subgroup of patients committing more errors agrees with studies showing that brain lesions and electrical stimulation of these tracts are involved in error commission. For instance, Han et al. (2013) demonstrated an association of lesions in the left ATR and left UNC with semantic deficits, while Mandonnet et al. (2019) showed that electrical stimulation of the striato-thalamic-cortical system, including the left ATR, evoked verbal perseverations. In addition, inclusion of the FAT in our study is notable, as this is a relatively new connective tract that is thought to have a key role in language (Catani et al., 2012). In a recent review, Dick et al. (2019) has proposed that the left FAT is involved in speech initiation, stuttering, lexical selection, and verbal fluency, and that the right FAT is involved in inhibitory control such as the stop of behavior. Accordingly, our data show that only the FAT had significantly higher MD values bilaterally in the AD subgroup with high error rate, which suggests that this specific pathway might be relevant for various processes subserving error commission in semantic fluency.

Moreover, the difference in MD values between the subgroup of patients with low-error rate and controls with high-error rate deserves attention. This patient subgroup had more deteriorated white matter of left UNC than the mentioned control subgroup. We consider this finding as relevant, since the UNC turns to be the only tract in our study showing significantly more white matter degeneration in the whole AD group. For this reason, we will discuss the importance of this tract in the commission of errors. On one hand, the literature highlights the role of the left UNC as a central pathway for semantic control and for general cognitive processes of inhibition and action selection (Duffau et al., 2009; Papagno, 2011). On the other hand, even if there is scarce number of studies addressing its role in error commission, there are data demonstrating that this tract is involved in the commission of semantic errors (Duffau et al., 2009; Sollmann et al., 2020). The UNC forms part of what is called an indirect way to the ventral semantic stream (Duffau et al., 2014). This pathway consists of the UNC linking the temporal pole with the inferior frontal gyrus *via* the pars orbitalis. It occurs that when electrically stimulated, areas conforming to the ventral semantic stream, semantic paraphasias (i.e., semantic errors) are evoked (Duffau et al., 2005). Thus, because the left UNC is the sole tract in which all the patients with AD show significantly higher MD values than the controls, it can be argued that this is the only tract involved in error commission in the whole AD group.

There are two remaining issues for discussion. First, we only obtained significant differences when contrasting the patient subgroups against the controls with high-error rate. We believe that these results are related to the composition of the older control subsamples. The subgroup of controls with high error rate comprises the majority of the healthy older participants (*n* = 14) with a higher number of males (*n* = 10), while the low-error control subgroup (total *n* = 9) has almost an equal number of males (*n* = 4) and females (*n* = 5). One would expect that older controls in the low-error subgroup may show unimpaired white matter integrity in all the tracts; therefore, significant differences should appear. However, this was not the case. Even though we controlled for gender in group comparisons at the second level of DTI analyses, we encountered a significant interaction with sex. This interaction yielded more asymmetric differences in MD values of all the three tracts in males than in females. Interestingly, control males in the high-error subgroup presented lower MDs in left hemisphere. Likewise, males in both subgroups of patients with AD showed this trend. Whether this asymmetry represents a real gender dimorphism or it relates to the peculiarity of our sample is difficult to establish, and future studies may pursue this line of inquiry with a larger number of participants. However, a definite contributing factor for the lack of group differences with the low-error rate subgroup of controls is that this subsample represents a group of individuals with more age-related deterioration on frontal white matter than the high-error subgroup, in spite of adequate VF performance. Such finding is not uncommon in research on aging, as many older participants considered cognitively normal present unnoticed clinical features similar to those of persons with dementia (Irwin et al., 2018).

The second issue is that group differences were found uniquely in the MD data. Usually, there is a tendency in aging studies in which white matter degeneration is expressed in terms of lower FA values coupled with higher MD values (Pini et al., 2016). However, this relationship is not always present (e.g., Huang et al., 2012; Chen et al., 2020). According to previous findings (Acosta-Cabronero et al., 2012), some DTI metrics are more sensitive to the early stages of AD, such as MD and axial diffusivity (DA). In our study, we confirm this assertion. Because MD is an average of the three eigenvectors calculated in DTI, increased MD values in our study cannot be translated into precise neurobiological changes; rather, these results indicate clear pathognomic signs of the selected frontal tracts, especially among patients committing the largest number of semantic VF errors.

### General Discussion

The present study was conducted to better understand the occurrence of semantic VF errors in normal aging and mild AD through a combined methodology. Although important research on the topic has long existed (see e.g., Fuld et al., 1982; Shindler et al., 1984; Hart et al., 1986), to our knowledge, no investigation has addressed the how and when of various types of errors in semantic VF, and has attempted to link this phenomenon to its neural correlates.

Four main findings arise from the present study. First, we found that error occurrence in semantic VF is triggered by semantic associations in all the participants disregarding their group affiliation. The issue of semantic relatedness of verbal error production has been acknowledged in studies where object naming or semantic priming is assessed (e.g., Vitkovitch and Rutter, 2000). Disclosing the nature of error occurrence in tasks of free recall such as VF is not obvious, since errors can be classified as unrelated words by a human coder. Hence by LDA, it was possible to appraise that an important proportion of errors are semantically associated with animal subcategories, at least at a lexical level even in patients. These findings point to the existence of relationships between the errors and subcategories that arise when thinking about animals and go beyond the strict inclusion of specimens of a given taxonomy. The results advocate, on one hand, for the importance of including errors in these types of analyses to accurately evaluate semantic dynamics in VF. On the other hand, the data inform us that the patients with mild AD are still preserving some degree of semantic network, which agrees with previous studies (Paganelli et al., 2003).

A second important finding relates to differences in error generation between the patients with AD and the healthy groups. We corroborated that perseverations were the sort of error most usually committed across the groups (Vitkovitch and Rutter, 2000). In healthy individuals of both age groups, perseverations were of little incidence but on most occasions, these were the only type of error committed. It is postulated that perseverations occurred because of reduction in language processing efficiency (Levelt, 1989), especially when people are under pressure to respond (Moses et al., 2004). Perseveration of words is more recurrent among healthy older participants who experience weakened working memory capacities (Ramage et al., 1999) and it is even more persistent in elders with mild AD (Kave and Heinik, 2017). Tentative explanations for the occurrence of perseverative answers in AD relate to impairment in lexical selection due to central executive dysfunction (Miozzo et al., 2013), attentional deficits (Rosen and Engle, 1997), and amnestic syndrome (Davis et al., 2002). Our findings agree with the proposal of Miozzo et al. (2013). However, they further suggest that perseverations during VF in mild AD are only an exacerbated deficit similar to the one occurring in normal aging, which does not represent a distinctive feature of the disease.

The third and probably most important finding of the present study concerns the occurrence of intrusions as a unique pathological feature of the AD group. Mostly, intrusions arise because of impaired semantic representations and impaired semantic knowledge (Paganelli et al., 2003). However, the mechanisms underlying intrusions are not solely related to memory retrieval (Shindler et al., 1984). Such assertion is supported by our time-course analysis, which points to deployment of different intertwined cognitive impairments all along VF performance. The impairments initially manifest as high incidence of intrusions due to semantic network abnormalities as well as deficient selection and judgment to generate appropriate responses. We consider that this initial stage poses a considerable effort in patients with mild AD as they try to activate memory search and word retrieval in a degraded semantic system (Paganelli et al., 2003). Consequently, the occurrence of intrusions takes place only during the first 15 s of the trial. From the second phase of the trial, the cognitive impairments that arise gradually are related to working memory deficits. These deficits are observed as lack of self-monitoring and difficulties to suppress already generated words, which emerge under conditions of fatigue or decreased attention (Hotz and Helmestabrooks, 1995). For this reason, a greater number of perseverations are observed at the end of the execution. Thus, in accordance with earlier suggestions (Davis et al., 2002), our findings reveal that perseverations and intrusions are consequences of different impaired mechanisms arising in different periods of task execution.

The fourth important finding in our study is regarding the confirmation of the hypothesis that white matter degeneration in frontal tracts is associated to error occurrence in mild AD. As expected, the selected frontal tracts (FAT, ATR, and UNC) were found to be significantly more deteriorated in the subgroup of patients committing high-error rate, and, specifically, they were more affected in the left hemisphere. This allows us to conclude that the selected tracts are of importance for the appearance of semantic VF errors in the patient group. In addition, the white matter integrity of the UNC also turned out to be significantly degraded among the subgroup of patients presenting low error rate, which suggests that deleterious changes in the microstructural properties of this specific tract underlie the commission of all type of errors (Von Der Heide et al., 2013).

Admittedly, the anatomical group differences encountered between the patient group and older controls can equally constitute just a coincidental event. However, the differences arise primarily in the left hemisphere of all the tracts, and a considerable body of data has reported these left hemisphere tracts as neural bases of general language functions (e.g., Chernoff et al., 2019; Dick et al., 2019) and language error commission (e.g., Han et al., 2013; Mandonnet et al., 2019). For this reason, we consider our findings rather connotative. Furthermore, we also corroborated that the healthy controls committing high-error rate showed significantly better tract integrity than the patients, which suggests that the appearance of intrusions in the patient group is related to higher deterioration of the mentioned tracts. It is worth noting that the vast majority of the healthy older adults committed very few errors. Thus, although we classified the older controls into “high and low-error subgroups,” these participants committed predominantly only few perseverations and nearly no intrusions. Taken together the above facts, we confirmed that the healthy older adults free of cognitive impairments who commit higher frequency of perseverations also showed deteriorations in specific frontal tracts (i.e., FAT) as compared to the younger individuals.

### Limitations

Some limitations of the present study should be acknowledged. First, we operated with a rather limited number of subjects, which were further reduced in the second stage of the study. Thus, the issue of low statistical power is present, and caution is demanded for generalization of the data. Furthermore, we did not conduct an analysis based on subtypes of perseverations and intrusions. As mentioned in the introduction, taxonomies for each type of error have been suggested (e.g., Sandson and Albert, 1984; Loewenstein et al., 1991). In turn, the various types of perseverations and intrusions are thought to reflect different cognitive impairments (Fischer-Baum et al., 2016). Thus, it would be advantageous that future research considers various subcategories of errors in a larger group of subjects to improve the understanding of the present findings. Finally, it is important to stress that degeneration of other connective pathways, such as those underlying the ventral semantic stream network (Duffau et al., 2014), might be equally involved in error commission during semantic VF. Nevertheless, the latter is not in disagreement with the view that deleterious changes in the ATR, FAT, and UNC are implicated in the commission of errors in mild AD.

## Conclusion

Our findings demonstrate that error production in mild AD during a 1-min trial of semantic verbal fluency follows a unique deployment of different error types that varies as a function of trial progression. This pattern of error occurrence clearly differentiates patients from healthy controls not only because of the way of deployment but also the presence of intrusions, which is a pathognomic trait proper to mild stages of AD (Loewenstein et al., 1991). Thus, our data document that intrusions and perseverations arise at different points in time, and that their emergence principally depends on executive functions and working memory impairments. Finally, our study strengthens the view that significant white matter deterioration of left frontal tracts exists in mild AD that corresponds to increased rate of semantic VF errors.

## Data Availability Statement

The datasets presented in this article are not readily available because of the restrictions imposed by the Regional Research Ethics Committee. Requests to access the datasets should be directed to CR-A, claudia.rodriguez-aranda@uit.no.

## Ethics Statement

The studies involving human participants were reviewed and approved by Regional Research Ethics Committee. The patients/participants provided their written informed consent to participate in this study.

## Author Contributions

CR-A and YI contributed to the conception and design of the study. CR-A collected data. KW and SJ recruited and assessed the patient group. CR-A and SC-C organized the MRI database. YI, CR-A, and SC-C performed the statistical analyses. YI, SC-C, and CR-A wrote the first draft of the manuscript. KW and SJ revised the intellectual content. All authors contributed to manuscript revision, and read and approved the submitted version.

## Conflict of Interest

The authors declare that the research was conducted in the absence of any commercial or financial relationships that could be construed as a potential conflict of interest.

## Publisher’s Note

All claims expressed in this article are solely those of the authors and do not necessarily represent those of their affiliated organizations, or those of the publisher, the editors and the reviewers. Any product that may be evaluated in this article, or claim that may be made by its manufacturer, is not guaranteed or endorsed by the publisher.
