# Supplementary material for: Evaluation of Error Production in Animal Fluency and Its Relationship to Frontal Tracts in Normal Aging and Mild Alzheimer’s Disease: A Combined LDA and Time-Course Analysis Investigation
Source: Front Aging Neurosci. 2022 Jan 12;13:710938. doi: 10.3389/fnagi.2021.710938 (PMC8790484; doi:10.3389/fnagi.2021.710938)
Supplement: Supplementary file 1 [file Data_Sheet_1.docx]

Supplementary Material

# Supplementary Material I

Correct response production by topic probability

Three of the topics showed the highest probabilities, especially among the older group and the AD patients in the total correct responses (Supplementary Figure 1, a and b). These were Topic 8, 2 and 14. For the young group the percentage of summed topic probabilities pertaining to these topics were 17.3%, 16.1%, and 13.5% respectively. Older adults produced 22.6%, 13.0, and 14.3% respectively for the mentioned topics, while the corresponding percentages for the AD group were 22.1%, 17.7% and 12.0%. An additional topic, namely Topic 9, attracted our attention, as it encompassed a good proportion of generated words by the older and patient groups. Percentages (see Supplementary Figure 1b) of summed topic probabilities for the latter topic were of 10.5% for younger adults, 9.8% for older participants and 14.4% for AD patients. Interestingly, the largest differences in percentage summed topic probabilities were of 5.3% in Topic 8 between the younger group and both older groups, and 3.8% in Topic 9 between the younger group and the patient group.


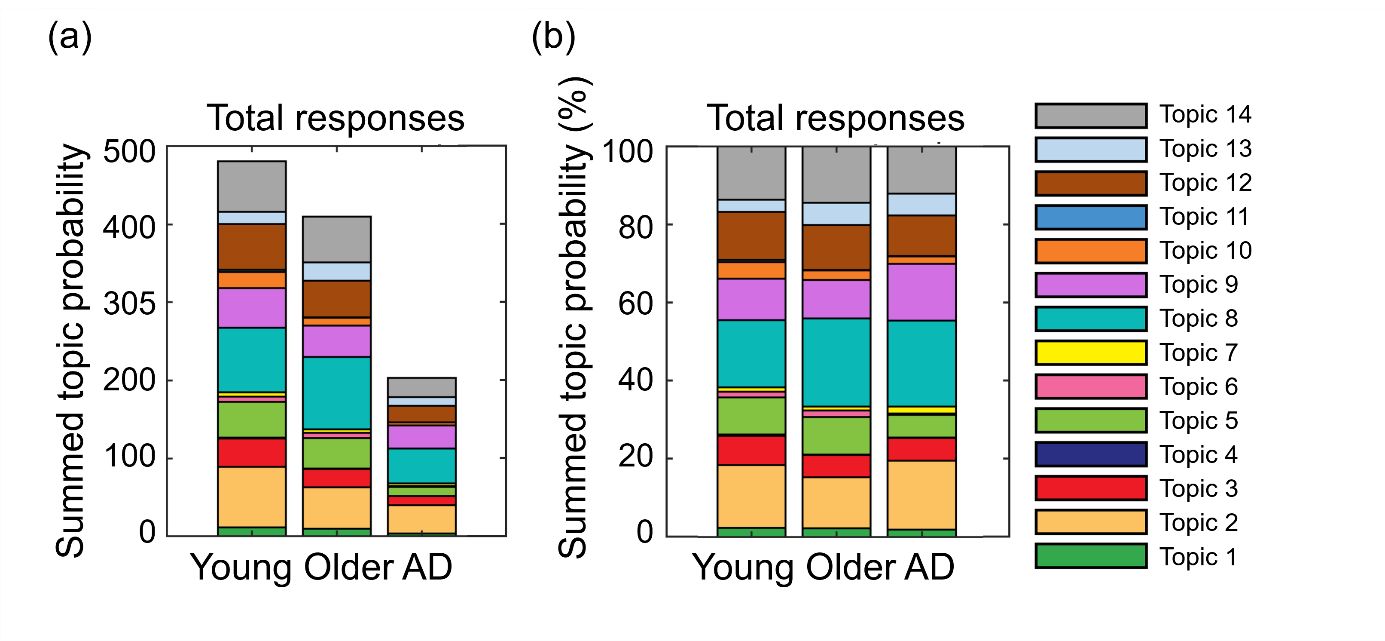


**Supplementary Figure 1.** The proportion of summed topic probabilities for each topic in total correct responses (a, b)

## Supplementary Figure 2

#
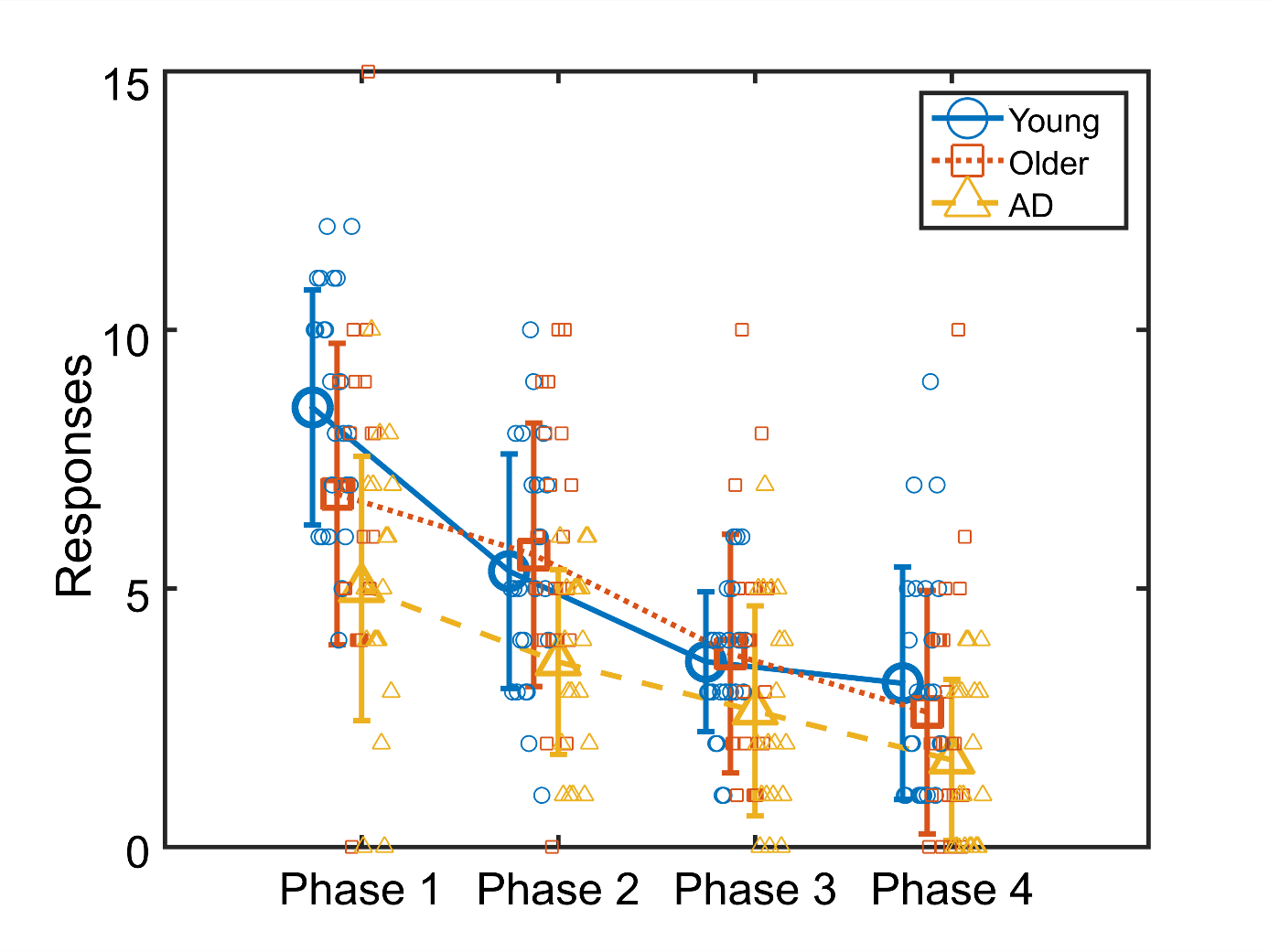


**Supplementary Figure 2.** Time course of the average number of responses (both correct responses and errors) in four phases. Small markers indicate individual data, and error bars indicate standard deviations.

# Supplementary Material II

Error commission of three groups: young, healthy older and mild AD

We present in this section relevant data of those individuals committing errors, which illustrates the reasons for exclusion of the younger group as well as the further subdivisions of the older groups. Supplementary Figure 3 shows the number of participants by group generating exact number of intrusions and perseverations. Nine out of the twenty-four younger adults committed one (n = 7) to two (n = 2) perseverations, which made 37.5% of the total sample. In the older group, fourteen participants committed the errors, which corresponds to 60.9% of this group. It is noteworthy mentioning that each of the fourteen controls committed either intrusions or perseverations. None of them committed both types of errors. As for the patient group, fifteen subjects corresponding to 78.9% of the group committed errors, whereof three of them generated both types of wrong responses.

The ratio of participants showing more than zero intrusions were 0/24, 2/23, and 8/20 in younger, older, and AD groups, respectively. A chi-square test showed a significant relation between the number of participants generating intrusions and group (χ^2^(2) = 14.81, *p* <.001, *w* = 0.47). The numbers of participants who showed more than zero perseverations were 9/24, 12/23, and 10/20 in younger, older, and AD groups, respectively. A chi-square test did not find a significant relation between the number of participants showing perseverations and group (χ^2^(2) = 2.00, *p =*.368, *w* = 0.17).

##
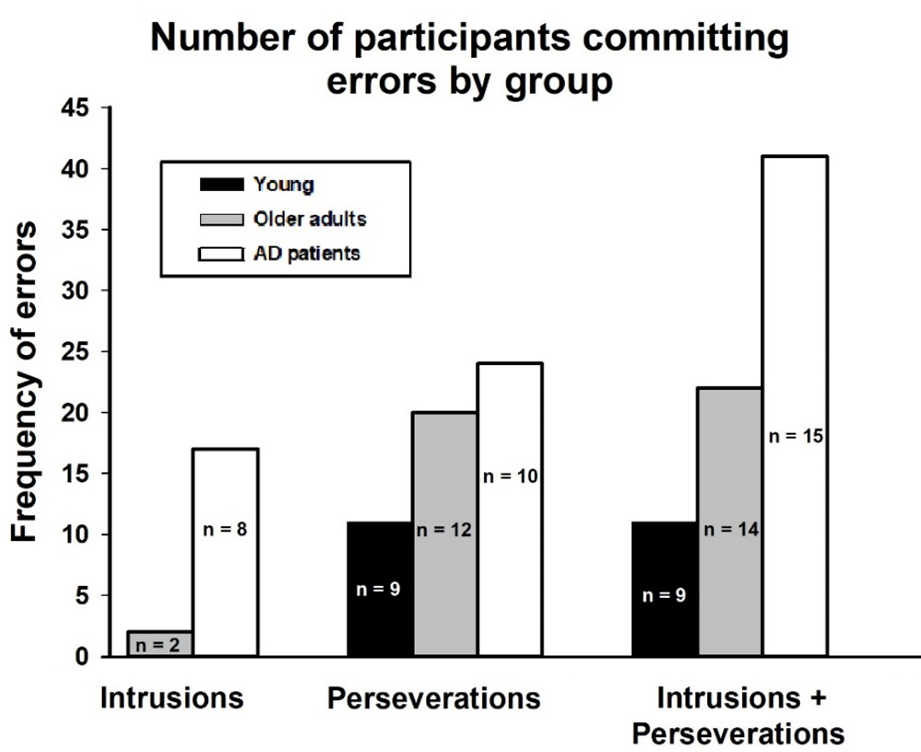


**Supplementary Figure 3.** Bars represent the effective number of errors commited by group. The number of actual participants generating each type of error is reported inside the bars.

# Supplementary Table 1

| Group differences in frontal tracts of young adults (Young), healthy older adults (Controls) and mild Alzheimer’s disease patients (Patients). | | | | | | |
| --- | --- | --- | --- | --- | --- | --- |
|  | **Young > Controls** | **Young > Patients** | **Controls > Patients** | **Controls > Young** | **Patients > Young** | **Patients > Controls** |
| Fractional Anisotropy |  |  |  |  |  |  |
| *Bilateral* |  |  |  |  |  |  |
| Anterior Thalamic Radiation | 0.005 | 0.005 | --- | --- | --- | --- |
| Frontal Aslant Tract | 0.003 | 0.003 | --- | --- | --- | --- |
| *Left Hemisphere* |  |  |  |  |  |  |
| Anterior Thalamic Radiation | --- | --- | 0.05 | --- | --- | --- |
| Uncinate Fasciculus | 0.005 | 0.005 | --- | --- | --- | --- |
| Mean Diffusivity |  |  |  |  |  |  |
| *Bilateral* |  |  |  |  |  |  |
| Frontal Aslant Tract | --- | --- | --- | --- | --- | 0.05 |
| *Right Hemisphere* |  |  |  |  |  |  |
| Anterior Thalamic Radiation | --- | --- | --- | --- | 0.05 | --- |
| Frontal Aslant Tract | --- | --- | --- | 0.03 | --- | --- |
| *Left Hemisphere* |  |  |  |  |  |  |
| Anterior Thalamic Radiation | --- | --- | --- | --- | --- | 0.001 |
| Frontal Aslant Tract | --- | --- | --- | --- | 0.03 | --- |
| Uncinate Fasciculus | --- | --- | --- | --- | --- | 0.03 |
| *Note:* p values for significant differences are shown; ---, non-significant. | | | | | | |
